# 913. Re-engagement using Historical Hepatitis C Antibody Results: Is it Worth the Effort?

**DOI:** 10.1093/ofid/ofab466.1108

**Published:** 2021-12-04

**Authors:** William Osborne, Noorann Sheikh, Gemma Botterill, Sally Bufton, David Mutimer, Mamoona Tahir, Sowsan F Atabani

**Affiliations:** 1 Royal Stoke and University Hospital, Stoke-on-Trent, England, United Kingdom; 2 University Hospitals Birmingham, Birmingham, England, United Kingdom; 3 West Midlands Hepatitis C ODN, Birmingham, England, United Kingdom; 4 University Hospitals Birmingham, UK, Birmingham, England, United Kingdom; 5 Public Health England, Birmingham, England, United Kingdom; 6 Public Health Laboratory Birmingham, Birmingham, England, United Kingdom

## Abstract

**Background:**

The World Health Organisation aim to eliminate hepatitis C (HCV) as a public health concern by 2030. One aspect of Public Health England’s (PHE) strategy to meet this target is to use historical surveillance data of anti-HCV positive patients identified by PHE to re-engage with offers of PCR testing and treatment if RNA-positive. Operational Delivery Networks (ODN) are responsible for enacting this initiative across 22 regions in England. We present an interim analysis and evaluation of the effectiveness of using this data to re-engage HCV-infected persons in the West Midlands ODN of England.

**Methods:**

A dataset of historical anti-HCV positive antibody patients provided to the West Midlands ODN by PHE was cross-referenced with HCV RNA data from 01/01/1996 to 01/01/2019 from 5 regional laboratories and regional treatment databases. If HCV RNA positive, letters were sent to the general practitioner and to the patient to invite them for further testing and, if necessary, treatment to achieve SVR. This received no additional funding or support and occurred in addition to the routine clinical workload.

**Results:**

From a dataset of 4,540 anti-HCV antibody results, 31.7% (n=1,440) had a PCR result: 48.1% (n=693) were PCR positive for HCV RNA with no evidence of cure. 693 letters were sent to GPs from Oct 2019 to Feb 2020 with responses from 14.2% (n=99). From July to Oct 2020 only 212 patient letters were sent (due to significant interruption due to the COVID-19 pandemic) and 11.3% (n=24) replied by May 2021. 17 presented for PCR testing and 4 were found to be viraemic. To date, one patient has achieved SVR and three have completed treatment awaiting SVR.

Re-Engagement Process

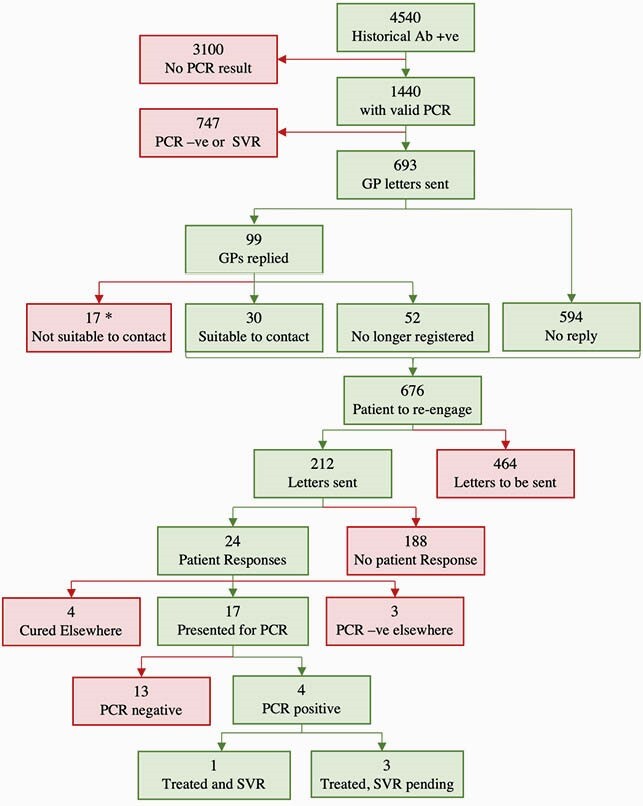

Flow diagram of re-engagement of patients with historical antibody-positive results for hepatitis C virus. * Of the 17 deemed not suitable to contact by the GP: 4 treated elsewhere, 3 had negative PCR elsewhere, 1 was unknown reason, 2 were under care of another hospital, 7 had died

**Conclusion:**

The use of historical anti-HCV antibody results to re-engage people into testing and treatment for hepatitis C in this format is low yield. Rollout was limited by ongoing clinical work and the COVID-19 pandemic. Dedicated time and resources with a less restrictive cohort might improve yields.

**Disclosures:**

**All Authors**: No reported disclosures

